# Enhancing biogas plant production using pig manure and corn silage by adding wheat straw processed with liquid hot water and steam explosion

**DOI:** 10.1186/s13068-017-0922-x

**Published:** 2017-11-09

**Authors:** Michał Gaworski, Sławomir Jabłoński, Izabela Pawlaczyk-Graja, Rafał Ziewiecki, Piotr Rutkowski, Anna Wieczyńska, Roman Gancarz, Marcin Łukaszewicz

**Affiliations:** 1 Department of Biotransformation, Faculty of Biotechnology, University of Wrocław, Fryderyka Joliot-Curie 14a, 50-383 Wrocław, Poland; 2 Department of Organic and Pharmaceutical Technology, Faculty of Chemistry, Wrocław University of Science and Technology, Wybrzeże Wyspiańskiego 27, 50-370 Wrocław, Poland; 3 Department of Polymer and Carbonaceous Materials, Faculty of Chemistry, Wrocław University of Science and Technology, Gdańska 7/9, 50-344 Wrocław, Poland

**Keywords:** Lignocellulosic biomass pretreatment, Steam explosion, Liquid hot water extraction, Methane production, Wheat straw

## Abstract

**Background:**

Pig manure utilization and valorization is an important topic with tightening regulations focused on ecological and safety issues. By itself pig manure is a poor substrate for biogas production because of its excessive nitrogen content relative to available organic carbon. Such substrate is alkaline, and methanogenesis can be suppressed, and so additional substrates with high organic carbon must be added. The most promising is straw, which is available from adjacent biogas plant cultures. However, the abundant lignocellulosic biomass of wheat straw undergoes slow decomposition, and only a fraction of the chemical energy can be converted into biogas; thus economical methods for pretreatment increasing bioavailability are sought.

**Results:**

A method was investigated to increase the methane yield in a full-scale plant for co-fermenting pig manure with corn silage, which was the default substrate in the original source reactors. Increased lignocellulosic bioavailability of wheat straw was achieved by combining liquid hot water (LHW) and steam explosion (SE). According to FT-IR analysis, the treatment resulted in hemicellulose hydrolysis, partial cellulose depolymerization, and lignin bond destruction. Low-mass polysaccharides (0.6 × 10^3^ g mol^−1^) had significantly higher concentration in the leachate of LHW-SE wheat straw than raw wheat straw. The methanogenic potential was evaluated using inoculum from two different biogas plants to study the influence of microorganism consortia. The yield was 24–34% higher after the pretreatment process. In a full-scale biogas plant, the optimal conditions were ~ 165 °C, ~ 2.33 MPa, and 10 min in LHW and ~ 65 °C and ~ 0.1 MPa for SE. The processes did not generate detectable inhibitors according to GC–MS analysis, such as furfural and 5-hydroxymethylfurfural.

**Conclusions:**

The LHW-SE combined pretreatment process increases the bioavailability of carbohydrates from wheat straw. The LHW-SE treated wheat straw gave similar biogas yields to corn silage, thus enables at least partial replacement of corn silage and is good for diversification of substrates. Surprisingly, microorganisms consortia from other biogas plant fed with other substrates may have higher efficiency in utilization of tested substrate. Thus, methanogenic consortia may be considered in the process of optimization at industrial scale. The efficiency was calculated, and the LHW-SE may be profitable at full industrial scale and further optimization is proposed.

## Background

The profitability of biogas plants in the European Union using biomass could be compromised without preferential regulations or market fluctuations, such as establishing low prices of green certificates [1]. Environmentally, the most advantageous option is processing organic waste in biogas plants instead of dedicated biomass grown on fields. However, the production capacity of biogas from waste may be too low for a biogas plant to be profitable. Therefore, there is a need for process optimization and the use of additional substrate [2, 3].

Biogas substrates vary in terms of the decomposition rate and in methane production yield. Therefore, a combination of feed additives and pretreatment methods should give the highest efficiency of a biogas production while reducing the decomposition time required for a substrate [4]. There are several methods of substrate pretreatment to improve the decomposition and methane yield [5]. However, for application on an industrial scale, these methods must be evaluated in terms of net energy gain and economic viability.

Pig manure alone is a poor substrate for biogas production, because of its excessive nitrogen content relative to available organic carbon. In addition high nitrogen content may result in toxic level of ammonia. Thus, additional substrates with high organic carbon must be added. The most promising is straw, which is available from adjacent biogas plant cultures [6]. However, the abundant lignocellulosic biomass of wheat straw undergoes slow decomposition, and only a fraction of the chemical energy can be converted into biogas. Increased lignocellulosic biomass conversion may be achieved by pretreatment methods such as liquid hot water (LHW) and steam explosion (SE) [7, 8]. The aim of this study is to-find alternatives and more economical methods for methane production for the Koczała full-scale biogas plant (POLDANOR; Poland) using pig manure and corn silage. For this purpose, the structural changes and the methanogenic potential in treated and untreated materials were investigated.

Novelty of our research results from the analysis of the impact of LHW-SE pre-treatment processing of wheat straw on its real biogas yield potential in the full-scale biogas plant with its comprehensive analysis. To date there are many studies which try to predict theoretically how particular substrate after pretreatment would behave in a full-scale biogas plant [9, 10] which are provided in small scale plants and laboratory studies. They point out the need to confront their assumptions with a full-scale plant results [11]. As shown in our research theoretical and real estimations are not consistent and the theoretical yield of biogas proved to be underestimated.

## Methods

### Raw material

Wheat straw (*Triticum aestivum* L.) was kindly supplied by the farms of Poldanor S. A. (Człuchów County, Pomorskie Voivodeship, Poland). The straw was dried in the field under the atmospheric conditions of a hot, dry summer and then stored in warehouse until use. The dry matter content of the wheat straw was 93.30% ± 0.20%. For LHW-SE pretreatment, light yellow, non-moldy wheat straw was chopped into approximately 10-mm pieces by a crop chopper (“DOZAMECH”, Odolanów, Poland). Recycled water was used in the LHW-SE pretreatment, which was obtained by mechanically squeezing post-fermentation sludge from a biogas plant.

### Liquid hot water–steam explosion pretreatment of wheat straw

LHW-SE pretreatment of the wheat straw was carried out in an industrial-scale combined installation (Koczała agricultural biogas plant, Poldanor S. A., Przechlewo, Poland). The concept of the plant construction is based on the general principles of the LHW and SE processes [12]. Briefly, the ground, dry wheat straw and recycled water were moved through a pipe reactor by a set of high-pressure pumps (2.33 MPa) with temperature maintained under the boiling point (~ 165 °C). The retention time in the pipe reactor was about 10 min to maintain the severity factor at the point where the inhibitors of the methane fermentation process are not produced, such as furfurals and 5-hydroxymethylfurfural (HMF) [7].

The wheat straw pulp then enters the decompression tank, where a rapid phase transition occurs. After expansion at 65 °C in the decompression tank, the wheat straw pulp is directly fed to the biogas plant. The liquid effluent (recycled water) from the biogas plant was used as a reaction medium in the LHW-SE process. The ratio of wheat straw to recycled water was between 20:1 and 23:1. The daily continuous LHW-SE processing plant processes 2300–3800 kg of wheat straw using 100–160 m^3^ of recycled water.

### Chemical characterization

The total solids (TS), volatile solids (VS), and ash contents were estimated according to the standard methods of the American Public Health Association [13] for preliminary characterization of the wheat straw, LHW-SE wheat straw, recycled water, and inocula used for biogas production. Fourier transform infrared (FT-IR) spectra of dry raw and LHW-SE pretreated wheat straw blades were obtained in the range of 400–4000 cm^−1^ on an FT-IR spectrophotometer (Bruker Vector 22 FT-IR) with a DTGS detector (Bruker, Germany) using a KBr disc containing 1% of the analyzed sample. The spectra were used to determine the changes in the functional groups that may have been caused by the pretreatment.

Size-exclusion chromatography (SEC) with an HPLC system was used to estimate the molecular mass of the water-soluble wheat straw products, compare them to the LHW-SE-processed pulp, and eliminate possible impurities from the recycled water. Water-soluble compounds from the wheat straw were isolated by maceration of 200 g of the ground wheat straw with 1000 mL of deionized water at room temperature for 7 days in the dark. The extract was then filtered through the Whatman filter paper to remove solids and evaporated under reduced pressure until dry.

The LHW-SE wheat straw pulp was centrifuged at 15,000×*g* for 10 min (Eppendorf Centrifuge 5804, Germany). The supernatant was collected and evaporated under reduced pressure until dry. The recycled water was also filtered through Whatman filter paper to remove some solid impurities and evaporated under reduced pressure until dry using a rotary evaporator. Each dry sample was dissolved in deionized water to obtain a concentration of 3 mg mL^−1^ and then centrifuged at 2000×*g* for 5 min. Each supernatant was filtered through a syringe filter with 0.45-µm pore size (Costar, Corning, NY, USA) and degassed before analysis.

For the chromatographic separation, tandem columns consisting of a Hema-Bio 300 and Hema-Bio 100 (Tessek, Czech Republic) were used with a total resolving power of mass in the range of 8 × 10^4^–6 × 10^5^ g mol^−1^. Deionized water was used as the eluent with flow rate of 0.6 mL min^−1^. The injection volume was maintained at 100 μL. The molecular mass and its distribution among the samples was analyzed based on saccharides and phenolics using an HPLC system (Gilson, Poland) equipped with a GX-271 Liquid Handler, a UV/VIS-152 detector at a wavelength of 270 nm, and a prepELS II evaporative light scattering detector. The temperature in the drift tube and the spray chamber were set as 45 and 10 °C, respectively. The molecular mass of the samples was estimated carried out using a calibration curve of dextran standards (7 × 10^4^, 2 × 10^5^, 5 × 10^5^ and 1 × 10^6^ g mol^−1^) (Sigma-Aldrich, Germany). The results were analyzed using Trilution LC software v2.1.

Some of the volatile products in LHW-SE wheat straw were analyzed using GC–MS to verify that the LHW-SE process was carried out under the conditions where the inhibitors of the methane fermentation process are absent. A pulp sample of LHW-SE wheat straw was centrifuged at 8000×*g* for 10 min to separate the straw blade fraction from the liquid suspension. The straw blades were dried at 37 °C for 14 days and then extracted according to a previously described method [14]. The drying was performed under vacuum in vacuum dryer [Binder VD 23 (E2.1), Germany] to avoid potential microbial degradation. The dry wheat straw blades (10.0 g) were macerated with 200 mL of chloroform and then 200 mL of methanol for 72 h each at room temperature. Each extract was filtered through Whatman filter paper and evaporated under reduced pressure until dry.

GC–MS analysis was performed according to Rutkowski and Kubacki [15]. In brief, each collected extract was dissolved in its previous solvent and analyzed using an HP6890 gas chromatograph equipped with an HP5973 mass selective detector and HP-5 ms column (25 m × 0.25 mm i.d., 0.25-µm film thickness, cross-linked 5% PH ME siloxane). The oven temperature program was 50–280 °C (4 °C min^−1^) after an initial 1 min isothermal period. The final temperature was kept for 10 min, and the flow rate of helium was 1 mL min^−1^. The inlet temperature was set at 260 °C. The sample injection was done in split mode (1:5). The mass spectrometer was set at an ionizing voltage of 70 eV with a mass range of *m/z* 15–450. Organic compounds were identified by comparing the mass spectra of the resolved components using NIST electronic-library search routines.

### Inocula and substrates

LHW-SE wheat straw and raw wheat straw were used as the initial substrates in laboratory-scale biogas production. Both were stored for 14 days before use in the dark in sterile, anaerobic, dry conditions in high-density polyethylene (HDPE) bags. The inocula of the methane reactors were taken from the Koczała biogas plant (KB) (Poldanor S. A. Przechlewo Poland), which processes pig manure and corn silage. A positive control was obtained from the Strzelin agricultural biogas plant (SB) (Südzucker Polska S.A. Strzelin Poland), which processes beet pulp. Both samples of inocula were taken 4 days before the experiment and stored at 20–37 °C in polyethylene jars.

### Experimental design of LHW-SE pretreated wheat straw methanogenic potential

Methanogenic potential tests were conducted similarly to Jabłoński et al. [16] with modifications. In the experiment 30 batch glass reactors with volumes of 1000 mL were used for measurements, five reactors for each experiment with dry wheat straw, LHW-SE pretreated wheat straw and without substrate as a reference sample. The reactors were loaded with inocula and operated for 28 days at a constant temperatures corresponding to the initial process carried out in the biogas plants which was 50 °C in KB and 39 °C in SB. At the beginning, 500 mL of inoculum was added into each bioreactor. Half of the bioreactors received the inoculum from SB and the other half received inoculum from KB. Next, 100 mL of LHW-SE wheat straw substrate was added to the first bioreactors containing SB inoculum (SB1). Similarly, 100 mL of LHW-SE wheat straw was added to bioreactors containing KB inoculum (KB1). For the third group of bioreactors with SB inoculum, 3.2 g of dry wheat straw and 100 mL of recycled water were added (SB2), and to the fourth group with KB inoculum received 3.2 g of the dry wheat straw and 100 mL of recycled water (KB2). The fifth group with SB inoculum was used as control probes and received 100 mL of distilled water (SBc). The sixth group of bioreactors with KB inoculum was the control mixtures and received 100 mL of distilled water (KBc).

The mass of dry wheat straw added to the digestate was chosen so that the initial amount of VS from the substrates would be equal. The samples were stirred manually just before the gas measurements. The amount of biogas produced from the biomass was calculated as the difference between the production in the sample bottles and the production in the blank bottles (without the addition of substrate). PVC urine bags of 2000 mL (Cezal, Poland) with drain valves connected to the reactors outlets were used as to collect the biogas. The volumes of the biogas produced were measured at established time intervals after 1, 2, 3, 4, 5, 7, 10, 12, 14, 17, 21 and 28 days. The gas samples were taken from the collecting containers from the dedicated outlet port using a 100-mL PVC syringe. The same operation was repeated for each reactor. Biogas volumes were calculated for the standard state (0.1 MPa).

### Koczała biogas plant basic characteristics

KB contains three fermentation tanks with a capacity of approximately 3010 m^3^ and two digestion tanks with a capacity of approximately 3990 m^3^. The temperature of the biogas production in the digesters is 50 °C, and the pH value is in the range of 7.45–7.60. The organic loading rate (OLR) [17] is 5.6 ${{\text{kg VS}} \mathord{\left/ {\vphantom {{\text{kg VS}} {\left( {{\text{m}}^{ 3} \;{\text{day}}} \right)}}} \right. \kern-0pt} {\left( {{\text{m}}^{ 3} \;{\text{day}}} \right)}}$, and the hydraulic retention time (HRT) is 31 days [17]. The maximum energy efficiency of the cogeneration engines (electric energy/thermal energy) of KB is 2126/2206 kWh, and the average methane concentration in the biogas is 51.5%. The engine efficiency is assumed to be 40%, and the methane energy value assumed to be 5.15 kWh m^−3^.

To theoretical biogas production was predicted as:

1 $$V_{\text{b}} = m_{\text{dm}} \times \;V_{\text{teo}} ,$$

where *V*_b_ is the theoretical biogas volume obtained from a substrate dry mass [m^3^], *m*_dm_ is the added mass of a particular substrate [t], and *V*_teo_ is the assumed biogas volume, which should be obtained from the substrate’s dry mass. *V*_teo_ is obtained from experiments or published sources. The sum of each type of a substrate for which the theoretical biogas volume was calculated as:

2 $$\mathop {\sum {V_{c} = V_{x} + V_{y} + V_{z} + V_{i} ,} }\nolimits$$

where *V*_*x*_, *V*_*y*_, *V*_*z*_, and *V*_*i*_ are theoretical biogas volumes calculated according to Eq. (1). Substrates used in the production of biogas were pig manure, corn silage and LWE-SE pretreated wheat straw. Recirculate counted as the fourth substrate.

### Energy balance calculation

Theoretical average daily energy gain [*G*] from LHW-SE pretreated WS was predicted as for period III:

3 $$G = M \cdot V \cdot P \cdot N,$$

where [*M*] is an average mass input of LHW-SE pretreated WS; [*V*] is an estimated actual LHW-SE pretreated WS biogas yield potential, [*P*] is an average methane concentration in the biogas; [En] is the methane energy value.

The final electrical energy [Ee] and thermal energy [Et] value was estimated from the engine efficiency [Ef]:

4 $${\text{Ee}} = G \cdot {\text{Ef}}$$

5 $${\text{Et}} = G - {\text{Ee}} .$$

### Statistical analysis

Experimental data were statistically analyzed with a Student’s *t* test with statistical significance level 0.05, implemented in Microsoft Office 2007.

## Results and discussion

**Fig. 1:**
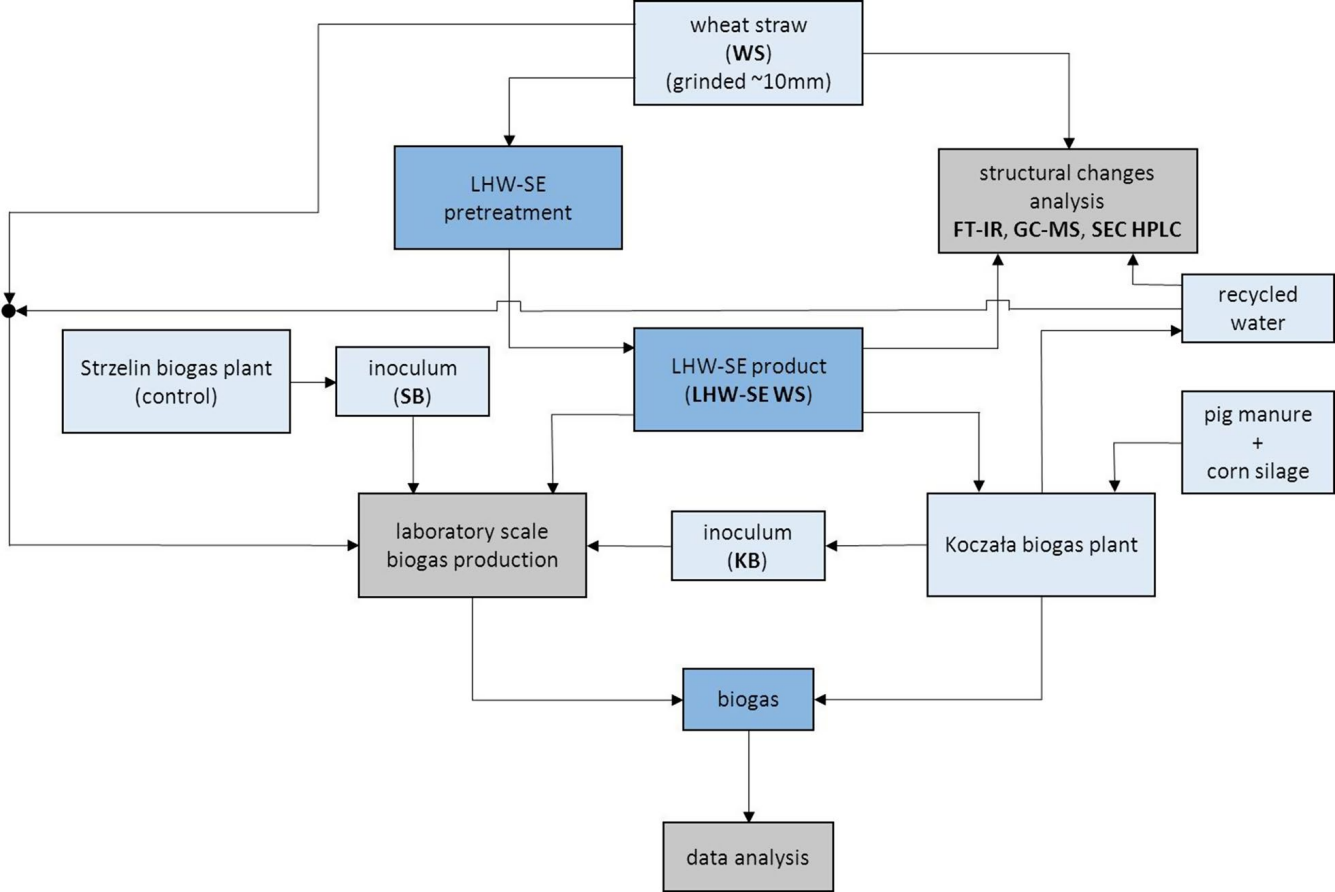
Schematic representation of the processes involved in the experimental setup

Raw, dry wheat straw (*Triticum aestivum* L.) was used as a material for pretreatment with LHW-SE to obtain a better substrate for biogas production. It was necessary to verify the laboratory results of the model process of biogas production with inoculum received from KB (Fig. 1) to assess the usefulness of the LHW-SE process in the biogas production process. HPLC, FT-IR, and GC–MS analyses were conducted to explain the influence of the pretreatment process on the wheat straw structure.

### Chemical characterization of wheat straw, LHW-SE wheat straw, and inocula

TS, ash, and VS amounts were estimated in the substrates, inocula, and recycled water (Table 1). Dry wheat straw contained high TS (93.3% w/w) and VS content (90.4% w/w), whereas the LHW-SE wheat straw had only 6.4% TS (w/w) and 5.4% VS (w/w). Both the wheat straw and LHW-SE wheat straw contained low amounts of ash (2.9 and 1.0% w/w, respectively). The Koczała inoculum used in the laboratory-scale experiments had 4.5% TS (w/w) and 3.6% VS (w/w), whereas the inoculum from the Strzelin plant used as a control contained less TS (3.1% w/w) and less VS (2.3% w/w). This difference might result from higher amount of the microorganism of KB or higher content of the undigested organic compounds. Both the KB inoculum and SB inoculum contained only 0.9% ash (w/w), while the recycled water was only 3.4% TS (w/w) and 0.8% ash (w/w). Recycled water contained (2.6% w/w) of VS. This may suggest a possible influence of using the recycled water as a medium in the KB biogas production process. To consider the possible impact on the results, recycled water was used in further analyses.

**Table 1:** TS, ash and VS used in the experi*ments of the biogas production*

	TS (w/w%)	Ash (w/w%)	VS (w/w%)
Dry raw WS	93.30 ± 0.20	2.94 ± 0.07	90.36 ± 0.27
LHW-SE WS	6.36 ± 0.21	0.98 ± 0.02	5.37 ± 0.23
Recycled water	3.39 ± 0.01	0.84 ± 0.02	2.55 ± 0.03
Inoculum of Koczała plant (KB)	4.50 ± 0.04	0.87 ± 0.01	3.62 ± 0.05
Inoculum of Strzelin plant (SB)	3.14 ± 0.02	0.89 ± 0.01	2.25 ± 0.03

Values are expressed as mean of five measurements ± SD

**Fig. 2:**
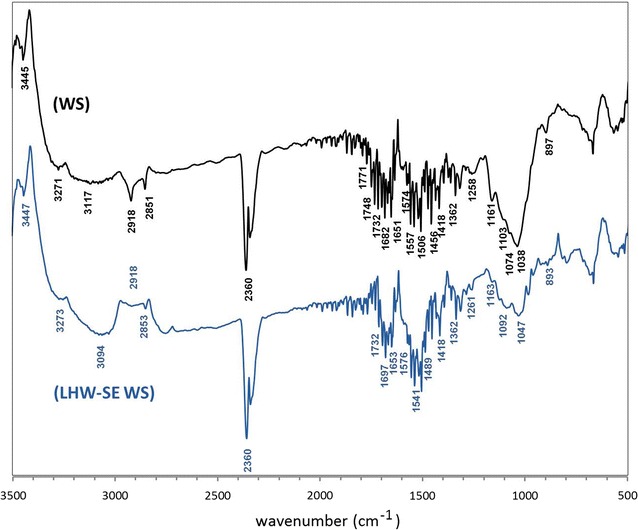
FT-IR spectra of wheat straw (WS) and its pretreated solids product (LHW-SE WS)

The finely ground samples of the wheat straw and LHW-SE wheat straw were analyzed using FT-IR spectroscopy (Fig. 2) to confirm that the pretreatment process caused structural changes in the plant material. The spectra indicated some similarities and differences. In the frequency region higher than 3000 cm^−1^, both spectra showed two wide bands. The first of them with a maximum occurred at 3272 cm^−1^ in both spectra, and the second bands occurred around 3094 and 3117 cm^−1^ for the wheat straw and for LHW-SE wheat straw, respectively. The second bands might correspond to the stretching vibrations *ν*(O–H) of phenolic groups of lignocellulosic structures as well as hydroxyl bonds from other saccharides in the cell walls of the wheat straw [18, 19]. Interestingly, after the pretreatment of plant tissues, the second band became more intense, which might indicate a higher concentration of free –OH from other carbohydrate compounds.

Another two bands of high intensity with maximums at 2918 cm^−1^ and at 2852 cm^−1^ were observed in both spectra due to stretching vibrations *ν*(C–H), but just the first of them was characteristic for −CH_3_ bonds, which are often present in the lignin network and as methyl esters of uronic acids in hemicelluloses. The second band confirmed the presence of (−CH_2_−) fragments from saccharide units [18, 19]. The stronger intensity of the first band in the wheat straw spectrum due to the symmetric stretching vibrations of (C–H) indicated a dominant amount of methyl bonds, which are probably present as the terminal ether bonds of the branched lignin structure [20]. These were almost not present in the LHW-SE wheat straw product. It might suggest that deesterification and perhaps even some delignification occurred during the pretreatment process [18].

Two sharp peaks centered around 2360 cm^−1^ were assigned to the characteristic vibration of the aromatic rings present in the lignocellulosic material [21]. In the range of 1800–2000 cm^−1^, a group of less intense signals was observed in both spectra. These peaks were overtones of aromatic rings, confirming the presence of a rich phenolic lignin structures. Guaiacyl–syringyl lignins (GS) in grass (including cereals) contain major amounts of structural elements derived from *p*-coumaryl alcohol, coniferyl alcohol, and sinapyl alcohol [20], as well as some polyphenolic acids as ferulic and *p*-coumaric acids [22, 23]. They create lignocellulosic macromolecular conglomerates with cellulose and with hemicelluloses full of carboxylic functional groups in esterified and free forms.

The presence of (C=O) bonds of the esterified types [19, 24, 25] were observed in both spectra as stretching vibration signals at 1748 and 1732 cm^−1^, but they were less intense in the LHW-SE wheat straw spectrum. In the contrast, a group of signals in the range of 1697–1634 cm^−1^ was more intense in the spectrum of the pretreated liquor product compared to the untreated wheat straw. This region typically shows stretching vibration signals of carbonyl groups from carboxylic bonds that are not esterified [19, 24, 25]. An additional band of the symmetric *ν*(C=O) stretching vibrations with a maximum at 1418 cm^−1^ was detected in both spectra. Another group of peaks in the range of 1580–1480 cm^−1^ was ascribed to the skeletal interactions of aromatic rings in lignin [26]. It became more intense after pretreatment of the wheat straw with the LHW-SE process. In summary, the characteristic features of delignification might be the increase in intensity of the general carbonyl absorbances in the range of 1770–1630 and around 1260 cm^−1^ [26].

Further bands indicated the presence of (C–H) bonds located at 1456 and 1373 cm^−1^, which were responsible for asymmetric and symmetric stretching interactions, respectively [23]. Moreover, the 1456/1506 cm^−1^ ratio was representative of the ratio of syringyl to guaiacyl (S/G) in lignin [27]. The lower S/G ratio in the LHW-SE wheat straw in comparison to the untreated wheat straw might suggest a loss of S monomers in the process of delignification. The other peaks might confirm this theory, where there was a less intense band for the *ν*(C–O–C) stretching vibrations typical for the syringyl rings at 1317 cm^−1^ in the spectrum of the pretreated liquor product. The band of the *ν*(C–O–C) stretching vibrations of the guaiacyl rings detected at 1261 cm^−1^ was shifted to 1258 cm^−1^ [23]. There was also lower intensity for the band of the *ν*(C–O–C) stretching interactions of *p*-coumaric ester groups typical for *p*-hydroxyphenyl guaiacyl and syringal (GSH) lignins detected at 1163 cm^−1^ [28].

FT-IR bands indicating the presence of polysaccharides were found at about 1074, 1038 and 1103 cm^−1^ [*ν*(C–O–C), *ν*(C–OH), and *ν*(C–C) of the saccharide rings], which were derived from cellulose and hemicelluloses [19, 29, 30]. After the LHW-SE process, the intensity of the signals in this region significantly decreased and shifted. In the spectrum of the LHW-SE wheat straw, signals were detected with maximums at 1126, 1092–1080, and 1047–1016 cm^−1^ [*ν*(C–O–C), *ν*(C–OH), and *ν*(C–C) of the saccharide rings]. This change might suggest a degradation of the polysaccharide network to shorter saccharide chains and monosaccharides.

In the anomeric regions of the FT-IR spectra of untreated wheat straw and LHW-SE wheat straw, the clear bands with low intensity at 897–893 cm^−1^ were attributed to the β-glycosidic linkages (1 → 4), which are especially characteristic of cellulose structure. For the α form, the bands typically occur at 837–840 cm^−1^ [30, 31]. The band of β bonds was much smaller in the spectrum of the pretreated liquor product. There might be few reasons, i.e. a weaker bonding dynamics of cellulose fibers due to the violation of the ordered crystal structure in the pretreatment process. Another reason of the lower intensity of this band might be the degradation of β-glycosidic bonds with polyphenolic compounds, where some loss of polyphenols in the pretreated liquor product by SEC analysis is confirmed.

**Fig. 3:**
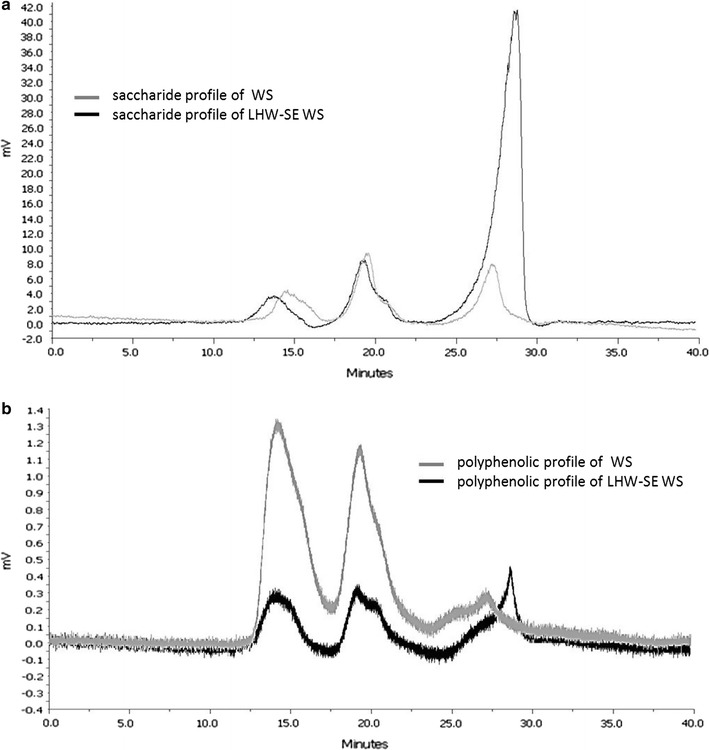
Size exclusion chromatography (SEC) analysis of water-soluble compounds. **a** Saccharide profile of wheat straw (WS) and of its pretreated liquor product (LHW-SE WS), identified by evaporative light scattering (ELS) detection method; **b** polyphenolic profile of wheat straw (WS) and of its pretreated liquor product (LHW-SE WS), where they were detected using UV–Vis detection system (*λ* = 270 nm)

SEC analysis of the water-soluble components was also performed using HPLC (Fig. 3a–c). Using a dual detection system comprising UV–Vis and electrospray light scattering (ELS) detectors, it was possible to detect some compounds with and without chromophore groups in their structures; i.e., polyphenolic glycoconjugates and pure saccharides. The water-soluble extracts of both wheat straw and LHW-SE wheat straw contained three fractions with polyphenolic–polysaccharide or oligosaccharide nature (Fig. 3, Table 2). SEC analysis of the wheat straw extract indicated peaks with molecular masses (*M*p) of ~ 1500 × 10^3^ g mol^−1^ (22.0% of the analyzed mixture), ~ 30 × 10^3^ g mol^−1^ (39.1%), and ~ 1–10 × 10^3^ g mol^−1^ (37.7%). In the chromatogram of the LHW-SE wheat straw extract, peaks with the following *M*p were detected: ~ 2 300 × 10^3^ g mol^−1^ (7.9%), ~ 30 × 10^3^ g mol^−1^ (16.3%), and ~ 0.2–1 × 10^3^ g mol^−1^. The last value is notable in that it represents as much as 75.6% of the analyzed mixture. Both chromatograms suggested a conjugate nature of the separated fractions, where saccharides were detected with similar retention time to polyphenolics, but the value was lower for the LHW-SE wheat straw. The water-soluble fractions of LHW-SE wheat straw contained much less polyphenolic compounds, and the average molecular mass of the last one suggested oligo- or even monosaccharide nature.

**Table 2:** Results of the water-soluble components of the LHW-SE WS pulp, and recycled water SEC HPLC analysis

Fraction no.	(WS)				(LHW-SE WS)			
	Average *M*p × 10^3^ g mol^−1^	%			Average *M*p × 10^3^ g mol^−1^	%		
		Polyphenolic part	Saccharide part	∑		Polyphenolic part	Saccharide part	∑
1	~ 1500	5.6	16.4	22.0	~ 2300	0.4	7.5	7.9
2	~ 30	3.5	35.6	39.1	~ 30	0.5	15.8	16.3
3	~ 1–10	6.6	31.1	37.7	~ 0.2–1	0.5	75.1	75.6
				98.8				99.8
Impurities from recycled water								
4	~ 1500	0.12	0.58	0.72	~ 1500	0.03	0.09	0.12
5	~ 160	0.08	0.42	0.48	~ 160	0.02	0.06	0.08
				1.20				0.20

After the pretreatment process the peak of monosaccharides with the longest retention time (Fig. 3a) significantly increased. Two peaks of macromolecular structures decreased, what is in the SEC chromatogram (Fig. 3b) well observed. In conclusion, the (LHW-SE WS) product is containing much more low molecular weight saccharides, which are the best absorbed carbon source for the microorganisms, to growth and development, what is inseparable from the increase in the productivity of biogas formation.

It was also necessary to check for possible impurities in the recycled water used in the pretreatment process. The results (Table 2) indicated that the use of compost water obtained from the post-fermentation process may have some influence on LHW-SE pretreatment process and methanogenesis in biogas plant. Small amounts of polysaccharides and polyphenolics such as glycoconjugates were found, but there were no monosaccharides. This suggests that the recycled water might be as good as tap water. In summary, the SEC analysis confirmed that the LHW-SE process leads to the hydrolysis of the polysaccharides contained in wheat straw into oligo- and monosaccharides.

Literature data indicate that inhibitors of the methanogenesis process such as furfural and its derivatives (i.e., HMF) might be produced in the LHW-SE pretreatment process [17]. GC–MS analysis was performed on extracts of the LHW-SE wheat straw, which were obtained using chloroform (extracted mass 4.7% w/w) and methanol (extracted mass 12.2% w/w). Concentrations of chloroform and methanol extracts used in GC–MS analysis were 0.0237 and 0.0610 g mL^−1^. A group of compounds were detected (Table 3), and some of them may have a positive influence on the methanogenesis process, such as carboxylic acids. These compounds are intermediate substrates and lead to the formation of acetate, carbon dioxide, and hydrogen, which are a crucial substrates for methanogenic archaea. These substrates may affect the overall high amounts of methane produce within the process. No common methanogenesis inhibitors were found [32] including furfural and its derivatives. Extracts of pretreated wheat straw solids did not show any typical inhibitors of the methane fermentation process, such as furfural and HMF, which indicates that the process was carried out under appropriate conditions, although there is still room for improvement. As we do not detect inhibitory compounds (ex. furfural) it should be possible to increase SF (severity factor) of the LHW-SE process [7].

**Table 3:** GC-MS analysis of the LHW-SE WS chloroform extract and methanol extract compounds

Signal no.	Retention time (min)	Compound name	Area (%)
		*Compounds in the chloroform extract*	
1	19.98	Dodecanoic acid	34.41
2	22.04	2-Pentadecanol	1.98
3	23.89	Hexadecanoic acid	4.63
4	27.90	Hexanedioic acid	6.07
5	31.09	1,2-Benzenedicarboxylic acid	13.17
		*Compounds in the methanol extract*	
1	5.41	Acetic acid	35.14
2	8.67	Propionic acid	2.61
3	9.36	Butanoic acid	3.07
4	12.55	Hexanoic acid	5.05
5	20.44	Ethylene	0.84
6	22.56	Pentadecanoic acid	2.98
7	23.90	Hexadecanoic acid	4.92
8	24.10	1,2-Benzenedicarboxylic acid	1.52
9	24.34	9,12-Octadecadienoic acid (*Z,Z*)	6.73
10	24.51	9,12,15-Octadecatrienoic acid	0.96
11	25.62	Oleic acid	15.80

### Laboratory-scale of biogas from LHW-SE wheat straw

**Fig. 4:**
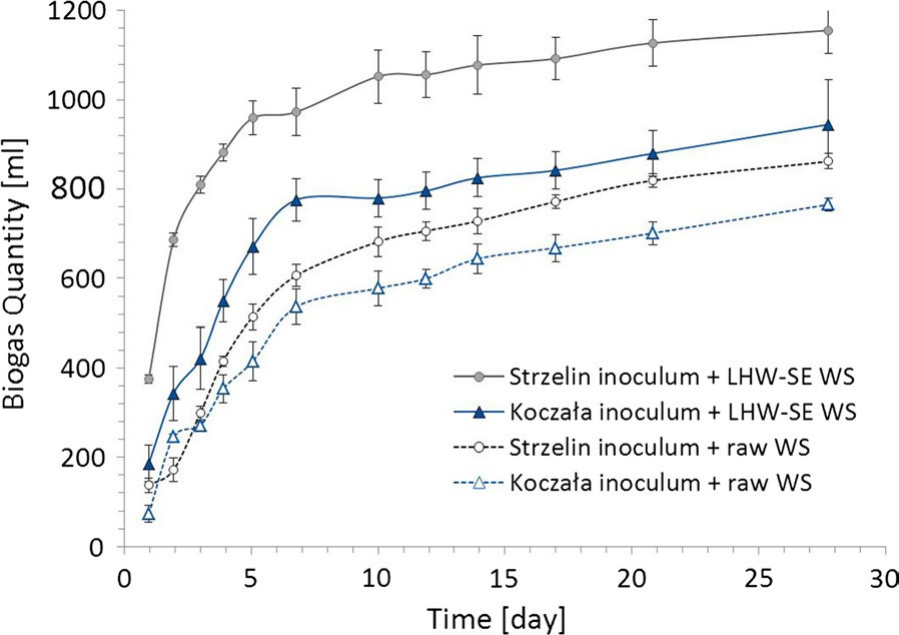
Biogas yield potential measurement of wheat straw (WS) and its pretreated product (LHW-SE WS)

The influence of the LHW-SE pretreatment of wheat straw was evaluated by biological tests of the methanogenic potential using two different methanogenic consortia: the inocula from the methane reactor of KB and SB. The SB inoculum was used as a control to see whether the different compositions of microorganisms have a significant effect on the amount of gas produced from the processed straw. The cumulative daily biogas production is presented in Fig. 4.

Experiments carried out in a laboratory. Data expressed in the cumulative average daily production after deduction of the control samples C.

The data show that the biogas production increased when using pretreated wheat straw as a raw material in comparison to raw wheat straw. The LHW-SE pretreatment improved the wheat straw decomposition and the methane yield by 24% when using inoculum from KB and by 35% when using inoculum from SB. Notably, the biogas production with the methanogenic consortium from SB was significantly higher than that obtained with the KB consortium. This may result from differences the in anaerobic digestion temperature, which was 50 °C for KB and 39 °C for SB. These temperatures could affect the species composition. Methanogenic species have a range spectrum of metabolic capabilities [33]. Thus, modification of the consortium could potentially be a good target for further increasing the process efficiency. Independently of the methanogenic consortium, the higher biogas yield after pretreatment suggests a change in the structure of the wheat straw, which contributed to the accelerated and increased production of biogas.

### Biogas and LHW-SE plants processing data

**Fig. 5:**
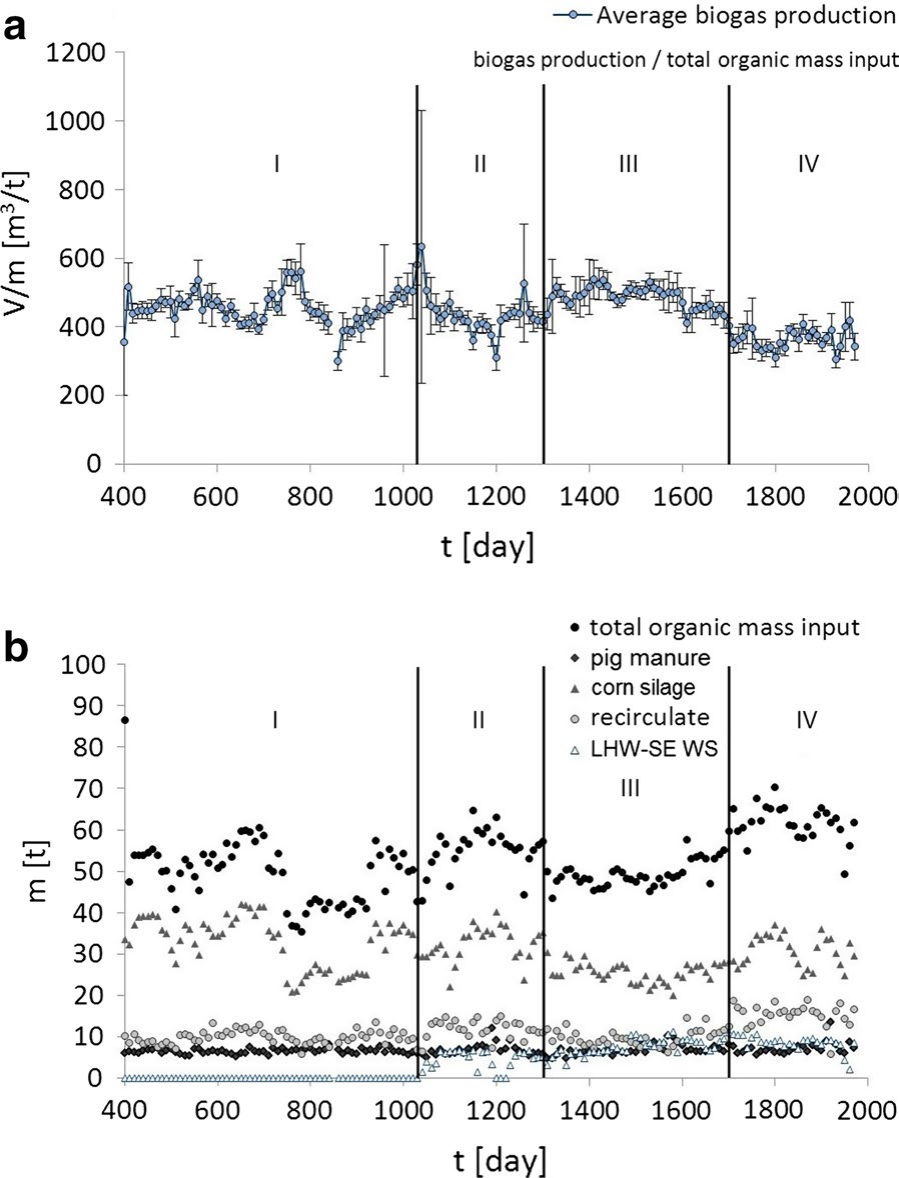
Processing data in Koczała biogas plant (KB) presented in the span of over 5 years. **a** The average biogas production on the total organic dry mass input. **b** Raw materials contribution in the total organic dry mass

**Fig. 6:**
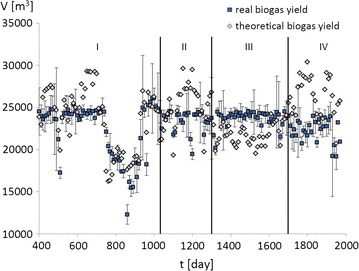
Correlation of real and theoretical biogas yield charts in Koczała plant over 1570 days of work

To estimate the actual impact of the LHW-SE pretreatment on methane fermentation in a biogas plant, the theoretical and real biogas yields were compared. Processing data from the KB biogas plant are presented for a span of over 5 years, including the averages of 10 days of sampling and standard deviations. The data presented in Figs. 5 and 6 include the average biogas yield in reference to the total organic dry mass (TOC) (Fig. 5a), the type and quantity of organic mass input (Fig. 5b), and actual and theoretically estimated biogas yield (Fig. 6).

The theoretical biogas yield was estimated by assigning a methanogenic potential to each independent substrate (Table 4). Observations started on the 400th day because many malfunctions occurred and the methanogenesis was not stable in the first year of operation which mainly resulted from pump failures, unsealing of high-pressure installations and clogging of pipes. The LHW-SE pretreatment plant was launched on the 1040th day, when observation period I ends and period II begins. During period I, a major malfunction occurred on the 750th day and lasted 200 days, during which the biogas yield dropped by half. This period enabled confirmation of the correlation between the theoretical biogas yield (for each biomass substrate except the pretreated wheat straw) and the real biogas yield. During period II, the LHW-SE pretreatment plant for wheat straw began operation and was stabilized.

**Table 4:** The average biogas yield produced from the dry organic mass

Raw material	Theoretical biogas yield (m^3^ t_dm_^−1^)
Pig slurry	320^a^
Corn silage	610^b^
Recirculate	150^c^
LHW-SE WS	350^c^/600^d^

^a^m^3^ t_dm_^−1^ of biogas yield from the dry organic mass depending on the literature data [4]

^b^m^3^ t_dm_^−1^ of biogas yield calculated, based on the methanogenic potential of recirculate measured in the experiments in the laboratory scale

^c^350 m^3^ t_dm_^−1^ of biogas yield calculated, based on the methanogenic potential of LHW-SE WS according to the laboratory data, measured in the experiments in the laboratory scale

^d^600 m^3^ t_dm_^−1^ of biogas yield obtained according to the biogas plant data

The biogas plant aims for an average biogas yield of ≈ 500 m^3^ t_dm_^−1^ in reference to TOC in methane fermentation, except in periods when the plant is overfed (Fig. 5a). Overfeeding occurs due to organic overload [16], when the amount of organic matter fed to the biogas plant exceeds the total degradation capacity of the microbes to produce biogas. In this case, Fig. 5b shows that the ratio of substrates changes because of the corn silage input increases together with the TOC. Despite the high input, the biogas yield drops due to overfeeding.

Figure 6 shows that the highest and most stable biogas production occurs in observation period III (between the 1300th and 1700th days), despite the lowest overall TOC and significant drop in corn silage input. The drop was slightly compensated by increasing the addition of LHW-SE wheat straw. The theoretical and real biogas yield in period III (Fig. 6) shows the actual long-term biogas yield was higher than the theoretically estimated yield for the first time, despite the low TOC and corn silage input. The only parameters that changed significantly during this time were the quantity and quality of the pretreated wheat straw. It was concluded that the laboratory data on the theoretical methanogenic potential underestimated the actual performance. The biogas potential estimated in the experiments was 350 m^3^ t_dm_^−1^ of biogas yield, but the value estimated from the theoretical and actual yield revealed 600 m^3^ t_dm_^−1^ of biogas yield from LHW-SE pretreated wheat straw. The difference between the laboratory-estimated and experimentally measured methanogenic potential of wheat straw may have resulted from the positive impact of the different substrates used in co-digestion [4].

Observation period IV includes an attempt to increase the biogas yield by increasing the input of corn silage and recirculated mass. However, the attempt failed and ended up in overfeed conditions. The biogas yield sudden decreases on the 1700th day, which lasted 270 days until the end of the experiment. The point with the biggest deviation occurred on day 1040, which corresponds to the launch of the LHW-SE pretreatment plant. The big deviation appeared because the digesters were not fed such for a few days before the launch. The big deviations on the 950th, 1260th, and 1320th days resulted from malfunctions in the biogas plant which mainly resulted from pump failures, unsealing of high-pressure installations and clogging of pipes.

In period III, the biogas yield of 600 m^3^ t_dm_^−1^ corresponds to the corn silage methanogenic potential. This observation suggests that the LHW-SE wheat straw could be a good substitute for corn silage, which is easily accessible and a cheap waste biomass material with the same methanogenic potential.

### Theoretical profitability of LHW-SE pretreatment process

Theoretical profitability was estimated based on the average plant energy consumption, data presented in Table 5. Complementing them with LHW-SE pretreated WS methanogenic potential, data from Table 4 and the input and output data from Figs. 5 and 6, we estimated theoretical net energy profit presented in Table 6.

**Table 5:** Average daily energy consumption in liquid hot water–steam explosion plant

Energy type	LHW-SE (kWh)	Mill (kWh)	Overall (kWh)
Electrical	568	59	627
Thermal	10,339	–	10,339
Σ	10,907	59	10,966

Average for 750 days of operation excluding malfunctions since LHW-SE launch

**Table 6:** Average theoretical daily energy net profitability from liquid hot water–steam explosion plant

Energy type	LHW-SE (kWh)
Electrical	15,300
Thermal	13,500
Σ	28,800

Although the theoretical profit seems to be large, unfortunately its potential has not been exploited. This was due to the continuous failure of the installation, the lack of potential heat energy buyers and the unstable process of methanogenesis in the biogas plant caused by overfeeding. That is why sometimes gross profit from using this type of plant was negative.

## Conclusions

This study confirmed the hypothesis that the LHW-SE combined pretreatment process increases the bioavailability of carbohydrates in wheat straw for methane fermentation microorganism consortia. The KB inoculum fed with pretreated wheat straw increased the methane yield by 24% in comparison to raw straw. Surprisingly, the SB inoculum produced biogas more efficiently, with 34% higher performance in comparison to the KB inoculum. The data obtained from the KB biogas plant before and after using the LHW-SE pretreated wheat straw suggest that it is good to diversify the substrates, which give similar biogas yields to corn silage. According to Jabłoński et al. [33], continuous-flow reactors are favored in one-step pretreatment processes because of their continuous procedure. However, they lead to major drawbacks of relatively low substrate concentrations and high energy demand for processing (due to pressure and heating in our case). The batch autoclave has an advantage because no substrate processing is necessary, and high solid-to-water ratios can be used. However, the decomposition of sugars can lead to undesired degradation products (furfural, HMF), insufficient lignin removal, and poor enzymatic digestibility. Rogalinski [34] proposed using a fixed-bed reactor that minimizes the disadvantages and enhances benefits of these two types of reactors. Combined processes of liquid hot water (LHW) and steam explosion (SE) could be considered as a good option for the green pretreatment of biomass. However, a new kind of plant should be developed while taking into account the minimization of heat and processing costs, as well as undesired degradation products with higher lignin degradation rates and enzymatic availability. The latest research indicates that the hydrothermal pretreatment of lignocellulosic biomass continues to be developed [35], and its profitability could still be increased.

## Abbreviations

ELS: evaporative light scattering
FT-IR: Fourier transform infrared spectroscopy
KB: the Koczała biogas plant
LHW-SE: liquid hot water–steam explosion
SB: Strzelin biogas plant
SEC: size exclusion chromatography
TOC: total organic dry mass
TS: the total solids
VS: volatile solids
WS: wheat straw
